# Supplementary material for: Establishing MS2–MCP-based single-molecule RNA visualization in Schizosaccharomyces pombe
Source: J Cell Sci. 2026 Jul 8;139(13):jcs264809. doi: 10.1242/jcs.264809 (PMC13380965; doi:10.1242/jcs.264809)
Supplement: Supplementary file 1 [file joces-139-264809_review_history.pdf]

Journal of
Cell Science

Establishing MS2-MCP-based single-molecule RNA visualization in *Schizosaccharomyces pombe*

Douglas E. Weidemann, Sarah C. Turner, Samir G. Chethan and Silke Hauf
DOI: 10.1242/jcs.264809

Editor: Megan King

Review timeline

Original submission:	18 February 2026
Editorial decision:	25 March 2026
First revision received:	2 June 2026
Accepted:	3 June 2026

Original submission

First decision letter

MS ID#: jcs.264809

MS TITLE: Establishing MS2-MCP-based single-molecule RNA visualization in *Schizosaccharomyces pombe*

AUTHORS: Douglas E. Weidemann; Sarah C. Turner; Silke Hauf

ARTICLE TYPE: Tools and Resources

Dear Silke,

We have now reached a decision on the above manuscript.

As you will see, the reviewers are enthusiastic about your manuscript and raise a small number of points that need to be addressed in a revision. I hope that you will be able to address these points because I would like to be able to accept your paper, which I should be able to assess without further review.

Reviewer 1

Weidemann et al. present an implementation of MS2-based single-molecule mRNA analysis in fission yeast. Although this approach is well established in many systems, it is not trivial to set up in a new organism. The authors do a thorough analysis of expression levels of the MS2 RNA-binding protein, and investigate variations on the RNA tag that it recognizes. By optimizing both elements of the system, they establish parameters that will be of great use to other workers in the field. The paper is well conceived and well written, and will be of broad interest, both to labs that want to use the technology in fission yeast and as an example of how it establish the technology in other systems.

I have only one minor suggestion.

Page 3, Line 53: Expand to read "single-molecule sensitivity in living cells has not yet".

Reviewer 2

© 2026. Published by The Company of Biologists under the terms of the Creative Commons Attribution License (https://creativecommons.org/licenses/by/4.0/).

In this manuscript from Weidemann et al, the authors develop the MS2 RNA imaging system in fission yeast. Optimizing this technology takes patience and iteration, and I applaud the authors for going through the steps in S. pombe. I agree it is a missing part of the arsenal for single-molecule gene expression studies. The main variables the authors have investigated are: expression levels, nuclear/cytoplasmic partitioning, MCP monomer/dimer and the fluorophore. In my experience, although this problem is never 'solved' - every gene and insertion site requires customization - it is useful to have a vetted starting point.

Major comment:
The authors have done many of the important controls. However, there are typically two types of assays for MS2 imaging: nascent transcription and individual mRNA imaging in the cytoplasm. The main concern is aggregates, which can look a lot like functional RNA spots. They need two additional controls. First, they need to do smFISH in the tagged strain. Although it can de difficult to count MS2 -GFP spots for RNA expression, the ideal comparison would be: 1) smFISH to the transcript in the untagged strain (i.e. cdc13), 2) smFISH to the transcript using same probes as in #1 but in the tagged train; 3) smFISH to the MS2 repeats in the tagged strain (the inter-stem loop linker sequences usually work best); 4) measure of GFP spots in the tagged strain with MCP. I would expect differences, but the numbers should be in the ball park. Second, they need to see fluctuations in the transcription signal over time, due either to stochastic fluctutations or transcription changes over the cell cycle. A signal which doesn't fluctuate is usually a give away for an aggregate. This time-dependent fluctuation is probably the most convincing in my experience that the MS2 is working as advertised.

First revision

Author response to reviewers' comments

We thank both reviewers for their assessment. We were delighted to read that they both found the results useful and well done. We have now:

- addressed the reviewers' comments, as explained below.
- deposited the relevant plasmids to Addgene and RIKEN.
- made additional minor text changes (tracked) for clarification.

Comments from the Reviewers:

Reviewer 1: Weidemann et al. present an implementation of MS2-based single-molecule mRNA analysis in fission yeast. Although this approach is well established in many systems, it is not trivial to set up in a new organism. The authors do a thorough analysis of expression levels of the MS2 RNA-binding protein, and investigate variations on the RNA tag that it recognizes. By optimizing both elements of the system, they establish parameters that will be of great use to other workers in the field. The paper is well conceived and well written, and will be of broad interest, both to labs that want to use the technology in fission yeast and as an example of how it establish the technology in other systems.

I have only one minor suggestion.

Page 3, Line 53: Expand to read "single-molecule sensitivity in living cells has not yet".
Has been changed. Thank you for spotting the inaccuracy.

Reviewer 2: In this manuscript from Weidemann et al, the authors develop the MS2 RNA imaging system in fission yeast. Optimizing this technology takes patience and iteration, and I applaud the authors for going through the steps in S. pombe. I agree it is a missing

© 2026. Published by The Company of Biologists under the terms of the Creative Commons Attribution License (https://creativecommons.org/licenses/by/4.0/).

part of the arsenal for single-molecule gene expression studies. The main variables the authors have investigated are: expression levels, nuclear/cytoplasmic partitioning, MCP monomer/dimer and the fluorophore. In my experience, although this problem is never 'solved' - every gene and insertion site requires customization - it is useful to have a vetted starting point.

Major comment:

The authors have done many of the important controls. However, there are typically two types of assays for MS2 imaging: nascent transcription and individual mRNA imaging in the cytoplasm. The main concern is aggregates, which can look a lot like functional RNA spots. They need two additional controls. First, they need to do smFISH in the tagged strain. Although it can de difficult to count MS2 -GFP spots for RNA expression, the ideal comparison would be: 1) smFISH to the transcript in the untagged strain (i.e. cdc13), 2) smFISH to the transcript using same probes as in #1 but in the tagged train; 3) smFISH to the MS2 repeats in the tagged strain (the inter-stem loop linker sequences usually work best); 4) measure of GFP spots in the tagged strain with MCP. I would expect differences, but the numbers should be in the ball park. Second, they need to see fluctuations in the transcription signal over time, due either to stochastic fluctutations or transcription changes over the cell cycle. A signal which doesn't fluctuate is usually a give away for an aggregate. This time-dependent fluctuation is probably the most convincing in my experience that the MS2 is working as advertised.

(1) smFISH

We have conducted smFISH experiments with the suggested controls, both for *mad2*-dark-EGFP-24xMS2 and *cdc13*-sfGFP-24xMS2 strains (**Fig. 4 and S6**). Overall, we find:

- A large fraction of **co-localization between EGFP and MS2 probes** for *mad2* (where we had probes for double-labeling available) (**Fig. 4B,C**). The small fraction of MS2 spots without corresponding EGFP spots can be explained by the dominant 5’ to 3’ RNA degradation in yeast (e.g., Grochowski et al., 2024). We also observed a small fraction of EGFP spots without a corresponding MS2 spot. Those could be the result of precocious transcription termination, of 3’ to 5’ degradation, or might be false-positives, since we observed a few false-positive signals with the EGFP probes (**Fig. S6B,C**).
- The **number of mRNA molecules per cell** obtained using coding-sequence or MS2 probes was highly similar. Furthermore, the mean RNA numbers per cell (2.3 for *mad3* and 27 for *cdc13*) were in line with the numbers we had previously observed for the RNA without MS2 tag (~ 2-3 molecules per cell for *mad2* (Esposito et al., 2022, Fig. 1E) and ~20 molecules per cell for *cdc13* (Weidemann et al., 2023, Fig. 6E)). In the current experiments, the mean mRNA number per cell for *cdc13* without MS2 tag was 27.5 (**Fig. S6D**), even closer to the number with MS2 tag. We suspect that the slightly higher number compared to earlier experiments is a consequence of using a different algorithm for counting (U-FISH vs. FISH-quant; U-FISH is more sensitive in our experience).

Counting spots in live-cell imaging, we also found a larger number of spots for *cdc13* than *mad2*. The numbers were in the same range as those obtained by smFISH (mean 2.97 for *mad3* and 16.72 for *cdc13*, **Fig. 4H**). The small difference for *cdc13* could be accounted for by occasional insufficient MCP binding to MS2 or by the challenges of imaging highly mobile RNAs in live cells.

(2) Transcription changes over the cell cycle

We have now additionally MS2-tagged the *rad21* gene, a cohesin subunit, which is expressed in a cell cycle-dependent manner. Our previous single-molecule mRNA FISH experiments showed that its mRNA numbers peak in long cells (Weidemann et al., 2023, Fig. 5B), which is consistent with the requirement for cohesin during S-phase. (In *S. pombe*, S-phase occurs in bi-nucleated, long cells). Live-cell imaging of *rad21*-24xMS2 reproduced this known expression pattern, with a peak of mRNAs

© 2026. Published by The Company of Biologists under the terms of the Creative Commons Attribution License (https://creativecommons.org/licenses/by/4.0/).

in long cells, just prior to septation and cell division (**Fig. 5**). This makes it unlikely that the observed spots are aggregates. Furthermore, the rapid increase and decrease in numbers (within 5 minutes) suggest that transcription and mRNA degradation dynamics are not strongly impaired by the MS2 tag.

All in all, we take these findings as evidence that spots observed in live-cell imaging are indeed mRNA molecules and largely represent full-length mRNA.

Second decision letter

MS ID#: jcs.264809R1

MS Title: Establishing MS2-MCP-based single-molecule RNA visualization in *Schizosaccharomyces pombe*

Authors: Douglas E. Weidemann; Sarah C. Turner; Samir G. Chethan; Silke Hauf

Article Type: Tools and Resources

Dear Silke,

I am happy to tell you that your manuscript has been accepted for publication in Journal of Cell Science, pending standard publication integrity checks.

© 2026. Published by The Company of Biologists under the terms of the Creative Commons Attribution License (https://creativecommons.org/licenses/by/4.0/).
